# Home Blood Pressure Control and Drug Prescription Patterns among Thai Hypertensives: A 1-Year Analysis of Telehealth Assisted Instrument in Home Blood Pressure Monitoring Nationwide Pilot Project

**DOI:** 10.1155/2021/8844727

**Published:** 2021-04-14

**Authors:** Anut Sakulsupsiri, Pairoj Chattranukulchai, Sarawut Siwamogsatham, Patchaya Boonchayaanant, Witthawat Naeowong, Aekarach Ariyachaipanich, Vorarit Lertsuwunseri, Voravut Rungpradubvong, Sudarat Satitthummanid, Sarinya Puwanant, Suphot Srimahachota, Wacin Buddhari, Smonporn Boonyaratavej, Surapun Sitthisook, Prapimporn Shantavasinkul, Peera Buranakitjaroen, Apichard Sukonthasarn, Somkiat Sangwatanaroj

**Affiliations:** ^1^Pharmacy Department, King Chulalongkorn Memorial Hospital, Bangkok 10330, Thailand; ^2^Division of Cardiovascular Medicine, Department of Medicine, Faculty of Medicine, Chulalongkorn University, King Chulalongkorn Memorial Hospital, Bangkok 10330, Thailand; ^3^Chula Clinical Research Center, Department of Medicine, Faculty of Medicine, Chulalongkorn University, Bangkok 10330, Thailand; ^4^Division of Endocrinology and Metabolism, Department of Medicine, Faculty of Medicine, Chulalongkorn University, King Chulalongkorn Memorial Hospital, Bangkok 10330, Thailand; ^5^Division of Hospital and Ambulatory Medicine, Department of Medicine, Faculty of Medicine, Chulalongkorn University, King Chulalongkorn Memorial Hospital, Bangkok 10330, Thailand; ^6^Division of Nutrition and Biochemical Medicine, Department of Medicine, Faculty of Medicine, Ramathibodi Hospital, Mahidol University, Bangkok 10400, Thailand; ^7^Division of Hypertension, Siriraj Hospital, Mahidol University, Bangkok 10700, Thailand; ^8^Thai Hypertension Society, Bangkok 10310, Thailand

## Abstract

**Background:**

Several interventions have been proposed to improve hypertension control with various outcomes. The home blood pressure (HBP) measurement is widely accepted for assessing the response to medications. However, the enhancement of blood pressure (BP) control with HBP telemonitoring technology has yet to be studied in Thailand.

**Objective:**

To evaluate the attainment of HBP control and drug prescription patterns in Thai hypertensives at one year after initiating the TeleHealth Assisted Instrument in Home Blood Pressure Monitoring (THAI HBPM) nationwide pilot project.

**Methods:**

A multicenter, prospective study enrolled treated hypertensive adults without prior regular HBPM to obtain monthly self-measured HBP using the same validated, oscillometric telemonitoring devices. The HBP reading was transferred to the clinic via a cloud-based system, so the physicians can adjust the medications at each follow-up visit on a real-life basis. Controlled HBP is defined as having HBP data at one year of follow-up within the defined target range (<135/85 mmHg).

**Results:**

A total of 1,177 patients (mean age 58 ± 12.3 years, 59.4% women, 13.1% with diabetes) from 46 hospitals (81.5% primary care centers) were enrolled in the study. The mean clinic BP was 143.9 ± 18.1/84.3 ± 11.9 mmHg while the mean HBP was 134.4 ± 15.3/80.1 ± 9.4 mmHg with 609 (51.8%) patients having HBP reading <135/85 mmHg at enrollment. At one year of follow-up after implementing the HBP telemonitoring, 671 patients (57.0%) achieved HBP control. Patients with uncontrolled HBP had a higher prevalence of dyslipidemia and greater waist circumference than the controlled group. The majority of uncontrolled patients were still prescribed only one (36.0%) or two drugs (34.4%) at the end of the study. The antihypertensive drugs were not uptitrated in 136 (24%) patients with uncontrolled HBP at baseline. Calcium channel blocker was the most prescribed drug class (63.0%) followed by angiotensin-converting enzyme inhibitor (44.8%) while the thiazide-type diuretic was used in 18.9% of patients with controlled HBP and 16.4% in uncontrolled patients.

**Conclusion:**

With the implementation of HBP telemonitoring, the BP control rate based on HBP analysis was still low. This is possibly attributed to the therapeutic inertia of healthcare physicians. Calcium channel blocker was the most frequently used agent while the diuretic was underutilized. The long-term clinical benefit of overcoming therapeutic inertia alongside HBP telemonitoring needs to be validated in a future study.

## 1. Introduction

Hypertension (HT) has remained the most common modifiable cardiovascular risk factor for many decades. Nevertheless, successful blood pressure (BP) control is observed in less than half of the patients worldwide [1–4]. There have been vast global disparities in strategies to overcome uncontrolled HT including raising HT awareness among healthcare personnel, increasing the availability of potent antihypertensive drugs, and addressing the therapeutic inertia [5–9]. Home blood pressure (HBP) monitoring has become a widely accepted method for assessing the response to antihypertensive medications and has the incremental benefit of enhancing BP control [10–12]. This improvement may be attributable to the diagnosis and treatment of masked uncontrolled HT; patients had better adherence to the medications and overcoming therapeutic inertia of healthcare providers. Recently, a telemonitoring technology with remote electronic BP data transfer has been introduced as part of the out-of-clinic BP monitoring [6, 12–14]. Thailand implemented this technology in its TeleHealth Assisted Instrument in Home Blood Pressure Monitoring (THAI HBPM) nationwide pilot project, which was initiated in 2016 aiming to engage hypertensive patients in the self-monitored BP program [15]. It was designed to be a proof-of-concept multicenter study using telemonitoring technology with a cloud-based transmission system. All of the HBP data were uploaded to the cloud and could be accessed at a central computer located at any clinics/hospitals participating in the project. The data was analyzed by trained healthcare providers and can potentially facilitate the titration or intensification of antihypertensive medications when the patients returned for their follow-up visit.

This study evaluated the achievement of HBP control and drug prescription patterns in Thai hypertensive adults at one year of follow-up after starting the Thai HBPM project.

## 2. Materials and Methods

### 2.1. Study Design and Participant Population

This study is a one-year follow-up analysis of the THAI HBPM project. The study design and method have been published [15]. Briefly, the THAI HBPM project was a prospective observational study conducted at 46 centers across all regions of Thailand (see Supplementary Material for the detail of all participating sites). This is Thailand's first nationwide project that promoted the use of HBP measurement using telemonitoring technology. This project assessed the prevalence and characteristics of each HT subtype defined by the mean clinic blood pressure (CBP) and mean HBP using this new technology. Treated or untreated hypertensive patients who were older than 18 years old and had been diagnosed with HT for more than 3 months based on CBP, without prior regular HBP monitoring, were enrolled in this project. The cross-sectional data analysis of the prevalence and characteristic of each HT subtype obtained from the first 7-day HBP records has been recently published [15].

The present analysis includes only treated hypertensive patients who had complete data at baseline and at one year of follow-up.

### 2.2. Clinic and Home Blood Pressure Measurements

The study's BP measurement method has been detailed elsewhere [15]. Briefly, trained healthcare professionals measured CBP of the patients using the validated sphygmomanometer, after 3 minutes of rest while in a sitting position. Clinical validation between CBP and HBP readings was performed at the clinic during the enrollment phase as per the standard recommendation [16].

HBP data were obtained using the same validated automated, oscillometric devices (Uright model TD-3128, TaiDoc Technology Corporation, Taiwan; see Supplementary Figure 1). Trained healthcare providers instructed the patients to perform self-measured HBP monitoring twice a day (within 1 hour after waking in the morning, before taking antihypertensive agents or having breakfast, and 30 minutes before going to bed) after 3 minutes of rest in a sitting position with two consecutive measurements, 1 minute apart for each recording. Blood pressure measurement continued for 7 days per month [17–19]. To avoid self-reporting bias, HBP readings were automatically recorded in the device's memory. All patients were informed to bring the HBPM device along with them to their appointed clinic visit. At each follow-up visit, all recorded HBP data were transferred from the devices via USB cable to the Windows-based computer at the participating hospitals. The data will then be automatically forwarded to the cloud storage through the internet-based transmission system. Patients are also able to upload the data at the local health-promoting clinic where the nurse case manager will provide assistance prior to their next physician's appointment.

When the HBP data had been uploaded, the physicians could view the result on the computer screen via Uright Telehealth web-based system and could print out the report if needed. The physicians were allowed to adjust medications according to the HBP result at each follow-up visit, which was scheduled at 1-to-4-month intervals on a real-life basis. In general, the widely accepted target BP for treatment is <135/85 mmHg for the home BP and <140/90 mmHg for the clinic BP [20].

### 2.3. Data Analysis

The patients were categorized into controlled and uncontrolled HBP groups according to their last average 1-week HBP reading at one year of follow-up (< or ≥135/85 mmHg) [19–21]. Patients who had complete 7-day HBP records at the end of follow-up will be included in the analysis. We discarded the measurements taken on the first day and used the mean value of all the remaining HBP records for the data analysis [15]. The patient characteristics (e.g., sex, age, waist circumference, body mass index, and comorbid metabolic diseases) were collected at baseline. The CBP data, HBP data, and antihypertensive regimens were collected at baseline and at every follow-up visit. All data were recorded in the Uright Telehealth web-based system.

Categorical variables were described as numbers and percentages of frequencies. Continuous variables were shown as mean values and standard deviation (SD). Chi-square test and ANOVA were used for the analysis of categorical and continuous variables, respectively. The independent *t*-test was used to examine the mean difference between groups. *p* value <0.05 was considered to be statistically significant. We used SPSS software version 24.0 (IBM, USA) for statistical analysis.

## 3. Results

### 3.1. Patient Characteristics

Patients from 46 hospitals across Thailand participated in the THAI HBPM project as previously reported [15]. Twelve patients were lost to follow-up and 1 patient died during the 1-year period after the project was launched. Thus, 1,177 patients were included in the final analysis. The mean (±SD) age was 58.9 ± 12.3 years, 59% were women, 22.5% had chronic kidney disease with GFR <60 mL/min/1.73 m^2^, and 13.1% had diabetes. The majority of patients were enrolled in primary care hospitals (82%). The overall mean baseline CBP was 143.9 ± 18.1/84.3 ± 11.9 mmHg with 503 (42.7%) patients having CBP reading <140/90 mmHg while the mean baseline HBP was 134.4 ± 15.3/80.1 ± 9.4 mmHg with 609 (51.8%) patients having HBP reading <135/85 mmHg. The average white-coat effect (systolic BP/diastolic BP difference between CBP and HBP) was 8.9 ± 15.1/4.3 ± 9.7 mmHg. Overall, the patients took a mean (±SD) of 1.9 ± 1.0 antihypertensive agents daily at enrollment. At one year of follow-up, the overall mean CBP was 141 ± 16.2/83.1 ± 10.8 mmHg while the mean HBP was 132.6 ± 15.0/79.1 ± 9.5 mmHg with the mean number of antihypertensive agents of 1.9 ± 1.2 (Table 1).

### 3.2. Achievement of Home Blood Pressure Control

From Table 1, there were 671 (57.0%) patients who achieved HBP target at one year with an average HBP of 122.6 ± 7.6/74.0 ± 6.1 mmHg compared to the patients with uncontrolled HBP who had an average HBP of 144.8 ± 12.1/84.9 ± 8.7 mmHg. There were 624 (53.0%) patients who attained CBP of <140/90 mmHg. The patients in the uncontrolled HBP group had a higher prevalence of dyslipidemia (54.5% vs. 48.6%; *p*=0.038) and greater waist circumference (89.7 ± 10.6 vs. 88.4 ± 10.0 cm; *p*=0.030) compared to the controlled group.

Table 2 shows the distribution of age groups and HBP control rate. Patients aged 70–79 years could achieve the HBP target of 67.2% compared to the patients who were 18–29 years that achieved the lowest proportion (47.1%).

### 3.3. Prescribing Patterns of Antihypertensive Medications

**Figure 1:**
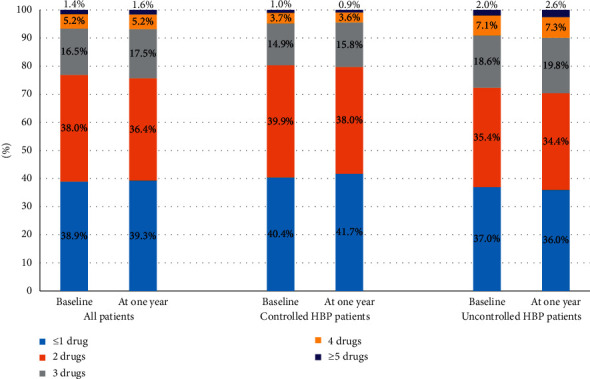
Number of antihypertensive medications prescribed at baseline and at one year of follow-up between the controlled and uncontrolled HBP groups at one year.

Figure 1 compares the number of antihypertensive medications prescribed at baseline and at one year of follow-up between the controlled and uncontrolled groups. Overall, the majority of the participants were on 1 or 2 types of antihypertensive drugs at both baseline (76.9%) and one year of follow-up (75.7%). Approximately 6.6% of the participants were on ≥4 types of antihypertensive drugs at baseline while the number marginally increased to 6.8% at one year of follow-up. The majority of the controlled participants were treated with 1 or 2 types of antihypertensive drugs at both baseline (80.3%) and one year of follow-up (79.7%). Among the participants with uncontrolled BP at follow-up, 70.4% of them were on 1 or 2 types of antihypertensive drugs, 19.8% of them on 3 drugs, 7.3% of them on 4 drugs, and 2.6% of them were treated with a combination of 5 or more drugs at one year of follow-up.

Among 567 (48.2) patients who had uncontrolled HBP at baseline, there were 124 (21.9) patients who had controlled HBP at the end of the study with a slight change in the number of medications from 2.03 ± 1.02 at baseline to 2.06 ± 1.05 at one year (*P*=0.64, Supplementary Figure 2). The antihypertensive drugs were not uptitrated in 136 (24%) patients with uncontrolled HBP who continued to receive the lowest dose at one year of follow-up.

**Figure 2:**
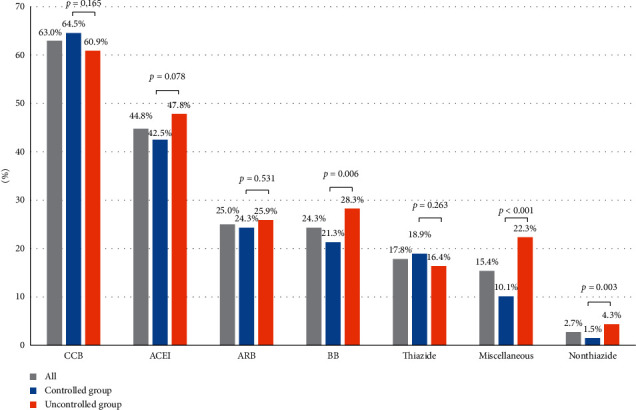
Prescribing frequency of each antihypertensive drug class at one year of follow-up. DM = diabetes mellitus; CCB = calcium channel blocker; ACEI = angiotensin-converting enzyme inhibitor; ARB = angiotensin receptor blocker; BB = beta-adrenergic blocker; Thiazide = thiazide-type diuretic (hydrochlorothiazide, chlorthalidone); nonthiazide = nonthiazide diuretic (amiloride, spironolactone, furosemide); miscellaneous = alpha-adrenergic blocker, hydralazine, clonidine, and methyldopa.

Figure 2 shows the prescribing frequency of each antihypertensive drug class at one year of follow-up. Overall, the most frequently used medication was calcium channel blocker (CCB, 63.0%) followed by angiotensin-converting enzyme inhibitor (ACEI, 44.8%), angiotensin receptor blocker (ARB, 25.0%), and beta-adrenergic blocker (24.3%). The thiazide-type diuretic was prescribed in 17.8% of the patients while 2.7% of the patients used nonthiazide diuretic. The beta-adrenergic blocker, nonthiazide diuretic, and miscellaneous drugs (i.e., alpha-adrenergic blocker, hydralazine, and methyldopa) were used more frequently in patients with uncontrolled HBP than the controlled group (28.3% vs. 21.3%; *p*=0.006, 4.3% vs. 1.5%; *p*=0.003, and 22.3% vs. 10.1%; *p*=0.006, resp.). The CCB was not only the most common single-agent regimen but also the most widely used drug in combination therapy for both groups. In a two-drug regimen, CCB plus ACEI was the most prescribed regimen; 106 patients (15.8%) with controlled HBP and 63 patients (12.5%) with uncontrolled HBP have prescribed this combination. In a three-drug regimen, the combination of CCB, ACEI plus beta-adrenergic blocker was the most commonly prescribed antihypertensive agents (Supplementary Table 1).

Amlodipine (88.5%), enalapril (99.2%), and losartan (91.2%) were the most common antihypertensive drugs used in its class (Supplementary Table 2).

Among 154 hypertensive diabetic patients, 43.9% and 34.4% of them were treated with ACEI and ARB, respectively (Supplementary Figure 3). The comparison of prescription pattern between primary and secondary/tertiary care hospitals are described in Supplementary Figure 4. Angiotensin-converting enzyme inhibitor was prescribed more in primary care hospitals (49.2% vs. 25.2%; *p* < 0.001) while ARB and beta-adrenergic blocker were more commonly used at secondary/tertiary care hospitals (42.2% vs. 21.1%; *p* < 0.001 and 34.9% vs. 21.9%; *p* < 0.001, resp.). Less than 20% of thiazide-type diuretic was prescribed at all levels of the hospitals.

## 4. Discussion

This study analyzed the achievement of HBP control and drug prescription patterns in Thai hypertensive adults at one year of follow-up after starting the THAI HBPM nationwide pilot project. The study used HBP of <135/85 mmHg as a treatment target. This goal has been supported by the recommendation from the national guideline [19] and has also been addressed in the Thai National Health Policy Program [20]. The result showed that the rate of successful HBP control was slightly increased from 51.8% at baseline to 57.0% at one-year follow-up after implementing the HBP telemonitoring while the rate of successful CBP control was increased from 42.7% at baseline to 53%. In comparison with prior studies in Thailand, the BP control rate ranged from 30% to 68%. This wide range was due to the different defined target BP, level of the participating hospital, and the method that was used to measure BP (i.e., CBP reading, HBP reading, or field BP measurement) [19, 22–24]. The data from HBP measurement is more predictive of long-term cardiovascular outcomes than CBP [13, 25] since it minimizes the white-coat effect that is commonly observed in CBP measurement [26]. The overall mean baseline HBP in this cohort was 134.4 ± 15.3/80.1 ± 9.4 mmHg while the mean baseline CBP was higher at 143.9 ± 18.1/84.3 ± 11.9 mmHg, confirming the presence of white-coat effect in real-life practice. Therefore, it is important to highlight that the BP control rate adjudicated based on HBP reading is considerably different from the studies that reported CBP data [27, 28].

In line with previous studies conducted in the Asian population [19, 22, 29], we found that the patients with uncontrolled BP had a higher prevalence of dyslipidemia and larger waist circumference than the controlled group.

The efficacy of telemonitoring to facilitate the BP control has been highlighted in several trials [11, 30, 31]. However, the present study showed that there was a small incremental change in the mean number of antihypertensive prescriptions compared between baseline (2.0 ± 1.0) and one year (2.1 ± 1.1) in patients with uncontrolled HBP. More specifically, approximately 70% of them were not prescribed an adequate guideline-recommended regimen of 3 or more medications at one year of follow-up. Not only that, but a large number of the patients that were initially prescribed monotherapy at baseline did not receive combination treatment after BP was not controlled during the follow-up period. Similarly, there was a slight change in the mean number of medications at the end of the study in patients with sustained HT at baseline. These imply that a therapeutic (or clinical) inertia might be the main reason for suboptimal BP control in this study. It is defined as the failure of healthcare providers to intensify or change antihypertensive medications appropriately when the target BP was not achieved [32, 33]. Ferrari et al. reported that primary care clinicians often do not intensify medication regimens [32]; even in the setting of a large, well-known clinical trial that followed a stringent protocol to achieve the targeted BP, therapeutic inertia was also detected [34]. There were many reasons why there was therapeutic inertia among the healthcare providers; it was shown that many physicians were not aware that the elevated HBP was considered “uncontrolled” whereas others may be overconcerned about the adverse effects of the medication [8, 32]. A wide variety of interventions to overcome therapeutic inertia have been reported in a systematic review [10]. The algorithm includes combination treatment, preferably a single-pill combination, which could be one of the solutions to solve therapeutic inertia by enhancing multidrug prescription [35].

In the present study, CCB was the most frequently used class of antihypertensive drugs in both groups, which accounted for more than 60% of all prescriptions followed by ACEI and ARB. This pattern is broadly accordant with the international guidelines [12, 14, 36] except for the use of the diuretic that was prescribed in less than one-fifth, especially in patients with uncontrolled HBP. More specifically, aldosterone receptor antagonist was prescribed in less than 1% of the patients with uncontrolled HBP despite having strong supporting evidence that it can be used as an adjunct treatment for patients with uncontrolled and resistant HT [12, 36, 37]. Contrary to the findings in early 2011 from Hypertension Audit in Clinical Practice Based in Thailand (HABIT) study [24], it showed that diuretic and CCB were the most common drugs prescribed to treat HT (45% vs. 49%). Even though it was also a nationwide study, however, it was conducted in 40 smaller level district hospitals where the newer antihypertensive medications were not yet widely available. In contrast, our study was initiated a decade later when ACEI and ARB are now widely available and affordable; thus, they are now more commonly prescribed to treat HT.

Renin-angiotensin system blockers are recommended in patients with diabetes coexisting with HT, especially when proteinuria or microalbuminuria is present [12, 36, 38]. In our study, approximately 78% of the patients with comorbid diabetes were treated with either ACEI or ARB. This result is in accordance with the percentage observed in real-world studies worldwide, which ranged from 58% to 89% depending on the level of the studied hospitals and the extent of diabetic complications of patients [38–41]. With regard to the prescription pattern based on the level of the hospital, the study showed that ARB and beta-adrenergic blockers were prescribed more frequently in secondary/tertiary care hospitals while ACEI was used more commonly in primary care centers. These findings are consistent with the results reported from recent studies conducted in Thailand [19, 22]. It is attributed to the fact that the higher-level facilities have patients with more complex comorbidities and have more access to newer antihypertensive drugs such as ARB.

There were some limitations in our study. First, the majority of patients were enrolled from primary care hospitals; hence, this may limit the generalizability of the results to larger care facilities. However, our findings can still represent the real-world situation in Thailand because the majority of hypertensive patients are followed at primary care centers [42]. Second, we did not assess the patients' adherence to antihypertensive drugs. The low compliance to treatment has played a major role in the poor rate of BP control in patients who have already been uptitrated [4]. Third, the prescription of antihypertensive drug(s) varied according to the patient's underlying disease; however, the prevalence of certain compelling comorbidities other than diabetes and chronic kidney disease was not collected. Forth, the selection bias should be mentioned since the rate of subjects with controlled HBP at baseline is pretty high (51%), suggesting that the white-coat effect is consequently high. Fifth, it could be argued that the physician may have considered patients who had normal CBP with elevated HBP (masked uncontrolled HT) as having an “optimal” BP status. As a consequence, they may use this as an excuse to not intensify the treatment regimen, mimicking therapeutic inertia [5]. This issue might be addressed in further research/survey to examine the reason for therapeutic inertia in Thai healthcare providers.

Despite the aforementioned limitations, the strength of our study is that this is the first multicenter study to examine HT control using telemonitoring technology across Thailand. We utilized the cloud-based HBP data transfer to overcome the self-reporting bias, which is a common limitation of conventional HBP measurement. Thus, this technological advancement provides us with more reliable HBP data. In addition, we utilized the average HBP readings to determine the achievement of BP control instead of using CBP. This could eliminate the white-coat effect and reflect the actual status of BP control.

## 5. Conclusion

Our findings suggest that the HBP control rate at 1 year after implementing HBP telemonitoring was still low. This is possibly attributed to the therapeutic inertia of healthcare physicians. Calcium channel blocker was the most frequently used antihypertensive drug while the diuretic was underutilized, especially in patients with uncontrolled HBP. Future studies are needed to determine the clinical benefit of overcoming therapeutic inertia alongside HBP telemonitoring for HT control in Thailand.

## Figures and Tables

**Table 1:** Demographics and patient characteristics.

Clinical characteristic	All *n* = 1,177	Controlled ^*γ*^ home BP group at one year *n* = 671 (57.0)	Uncontrolled ^*∗*^ home BP group at one year *n* = 506 (43.0)	*p* value
Age (year)	58.9 ± 12.3	59.3 ± 12.1	58.4 ± 12.6	0.236
Female	699 (59.4)	395 (58.9)	304 (60.1)	0.675
Diabetes	154 (13.1)	82 (12.2)	72 (14.2)	0.092
Dyslipidemia^$^	602 (51.1)	326 (48.6)	276 (54.5)	**0.038**
Follow-up at primary care hospital	959 (81.5)	544 (81.1)	415 (82.0)	0.680
Body mass index (kg/m^2^)	26.5 ± 4.9	26.3 ± 4.9	26.8 ± 4.9	0.102
Waist circumference (cm)	89.0 ± 10.3	88.4 ± 10.0	89.7 ± 10.6	**0.030**
Chronic kidney disease with eGFR < 60 mL/min/1.73 m^2†^	265 (22.5)	144 (21.4)	121 (23.9)	0.374

Hypertension status at baseline				
(i) Clinic BP				
Systolic BP (mmHg)	143.9 ± 18.1	139.0 ± 16.8	150.5 ± 17.6	**<0.001**
Diastolic BP (mmHg)	84.3 ± 11.9	81.8 ± 11.0	87.6 ± 12.3	**<0.001**
<140/90 mmHg	503 (42.7)	377 (56.1)	126 (24.9)	**<0.001**
(ii) Home BP				
Systolic BP (mmHg)	134.4 ± 15.3	126.7 ± 11.1	144.5 ± 14.1	**<0.001**
Diastolic BP (mmHg)	80.1 ± 9.4	76.2 ± 7.3	85.2 ± 9.3	**<0.001**
<135/85 mmHg	609 (51.8)	547 (81.5)	62 (12.3)	**<0.001**
(iii) Number of antihypertensive medications	1.9 ± 1.0	1.9 ± 0.9	2.0 ± 1.0	**0.004**

Hypertension status at one year of follow-up				
(i) Clinic BP				
Systolic BP (mmHg)	141.1 ± 16.2	132.8 ± 14.6	150.3 ± 15.4	**<0.001**
Diastolic BP (mmHg)	83.1 ± 10.8	79.1 ± 9.9	87.0 ± 11.3	**<0.001**
<140/90 mmHg	624 (53.0)	524 (78.1	100 (19.8)	**<0.001**
(ii) Home BP				
Systolic BP (mmHg)	132.6 ± 15.0	122.6 ± 7.6	144.8 ± 12.1	**<0.001**
Diastolic BP (mmHg)	79.1 ± 9.5	74.0 ± 6.1	84.9 ± 8.7	**<0.001**
(iii) Number of antihypertensive medications	1.9 ± 1.2	1.9 ± 1.5	2.1 ± 1.1	**< 0.001**

Values are presented as means ± SD, number (%). BP = blood pressure. ^*γ*^Controlled home BP group: participants who had home BP data at one year of <135/85 mmHg.  ^*∗*^Uncontrolled home BP group: participants who had home BP data at one year of ≥135/85 mmHg. ^$^Dyslipidemia was defined as the total blood cholesterol level greater than 200 mg/dL, or low-density lipoprotein cholesterol level greater than 130 mg/dL, or fasting triglycerides level greater than 150 mg/dL; or using lipid-lowering drugs. ^†^Estimated glomerular filtration rate (eGFR) calculated by the Modification of Diet in Renal Disease (MDRD) study equation.

**Table 2:** Home blood pressure control rate for different age groups^†^.

Age group (year)	Number	Controlled home BP ^*∗*^, *n* (%)
18–29	17	8 (47.1)
30–44	127	71 (55.9)
45–59	461	257 (55.7)
60–69	348	192 (55.2)
70–79	183	123 (67.2)
≥80	41	20 (48.8)
Total	1,177	671 (57.0)

BP = blood pressure.  ^*∗*^Controlled home BP: patients who had home BP data at one year of <135/85 mmHg. ^†^Age group is stratified according to the 5^th^ Thai National Health Examination Survey 2015.

## Data Availability

The data used to support the findings of this study are available from the corresponding author upon request.

## Supplementary Materials

Supplementary Materials Supplementary list of all 46 participating sites. Supplementary Figure 1: specifications of the oscillometric Home Blood Pressure Monitoring device used in the project (Uright model TD-3128, TaiDoc Technology Corporation, Taiwan). Supplementary Figure 2: number of antihypertensive medications prescribed at baseline and at one year of follow-up between the controlled and uncontrolled HBP groups at baseline. Supplementary Figure 3: prescribing frequency of each antihypertensive drug class at one year of follow-up in patients with and without diabetes. Supplementary Figure 4: prescribing frequency of each antihypertensive drug class at one-year follow-up according to the level of the hospital. Supplementary Table 1: the first three most prescribed regimens at one-year follow-up. Supplementary Table 2: prescribing frequency of each antihypertensive drug at one-year follow-up.
