# Supplementary material for: Home Blood Pressure Control and Drug Prescription Patterns among Thai Hypertensives: A 1-Year Analysis of Telehealth Assisted Instrument in Home Blood Pressure Monitoring Nationwide Pilot Project
Source: Int J Hypertens. 2021 Apr 14;2021:8844727. doi: 10.1155/2021/8844727 (PMC8060083; doi:10.1155/2021/8844727)
Supplement: Supplementary Materials — Supplementary list of all 46 participating sites. Supplementary Figure 1: specifications of the oscillometric Home Blood Pressure Monitoring device used in the project (Uright model TD-3128, TaiDoc Technology Corporation, Taiwan). Supplementary Figure 2: number of antihypertensive medications prescribed at baseline and at one year of follow-up between the controlled and uncontrolled HBP groups at baseline. Supplementary Figure 3: prescribing frequency of each antihypertensive drug class at one year of follow-up in patients with and without diabetes. Supplementary Figure 4: prescribing frequency of each antihypertensive drug class at one-year follow-up according to the level of the hospital. Supplementary Table 1: the first three most prescribed regimens at one-year follow-up. Supplementary Table 2: prescribing frequency of each antihypertensive drug at one-year follow-up. [file 8844727.f1.docx]

**Supplementary List of all 46 participating sites**

Vajira hospital; Bangkok (Peth Rod-aree), Prapokklao hospital; Chanthaburi (Wanna Chamjamrat), Uttaradit hospital; Uttaradit (Phatcharee Phengsupun), Maemoh hospital ; Lampang (Atitaya Churdchom), Mae On hospital; Chiang Mai (Linda Inphom), Banhong hospital; Lamphun (Pattaraporn Wongyai), Chiangkong Prince Crown hospital; Chaing Rai (Sukanya Sriprapaporn), Laplae hospital; Uttaradit (Chonlachab Jundoung), Aoluk hospital; Krabi (Chanisara Chaisiri), Pakpayun hospital; Phatthalung (Pimprapai Buakeaw), Khokcharoen hospital; Lopburi (Nuntawan Khwansuk), Chaiyo hospital; Angthong (Kunyarat Thongsod), Doembangnangbuat hospital; Suphan buri (Orachorn Panich), Phanomsarakham hospital; Chachoengsao (Dungmanee Wiyathus), Wapipathum hospital; Maha Sarakham (Benjaporn Intakornudom), Nawa hospital; Nakhon Phanom (Areerath Phaengyod), Dokkhamtai hospital; Phayao, Tha Wang Pha hospital; Nan, Song hospital; Phrae, Bang Pahan hospital; Ayutthaya, Mae Ra Mard hospital; Tak, Sawang Arom hospital; Uthai Thani, Thalang hospital; Phuket, Ban Na San hospital; Surat Thani, Pathio hospital; Chumphon, Bannang Sata hospital; Yala, Prajan Takam hospital; Prachinburi, Angthong hospital; Angthong, Bo rai hospital; Trat, Ko Sichang hospital; Chonburi, Bang Bo hospital; Samut Prakan, and King Chulalongkorn Memorial hospital; Bangkok

**Supplementary Figure 1:** Specifications of the oscillometric home blood pressure monitoring device used in the project (Uright model TD-3128, TaiDoc Technology Corporation, Taiwan).


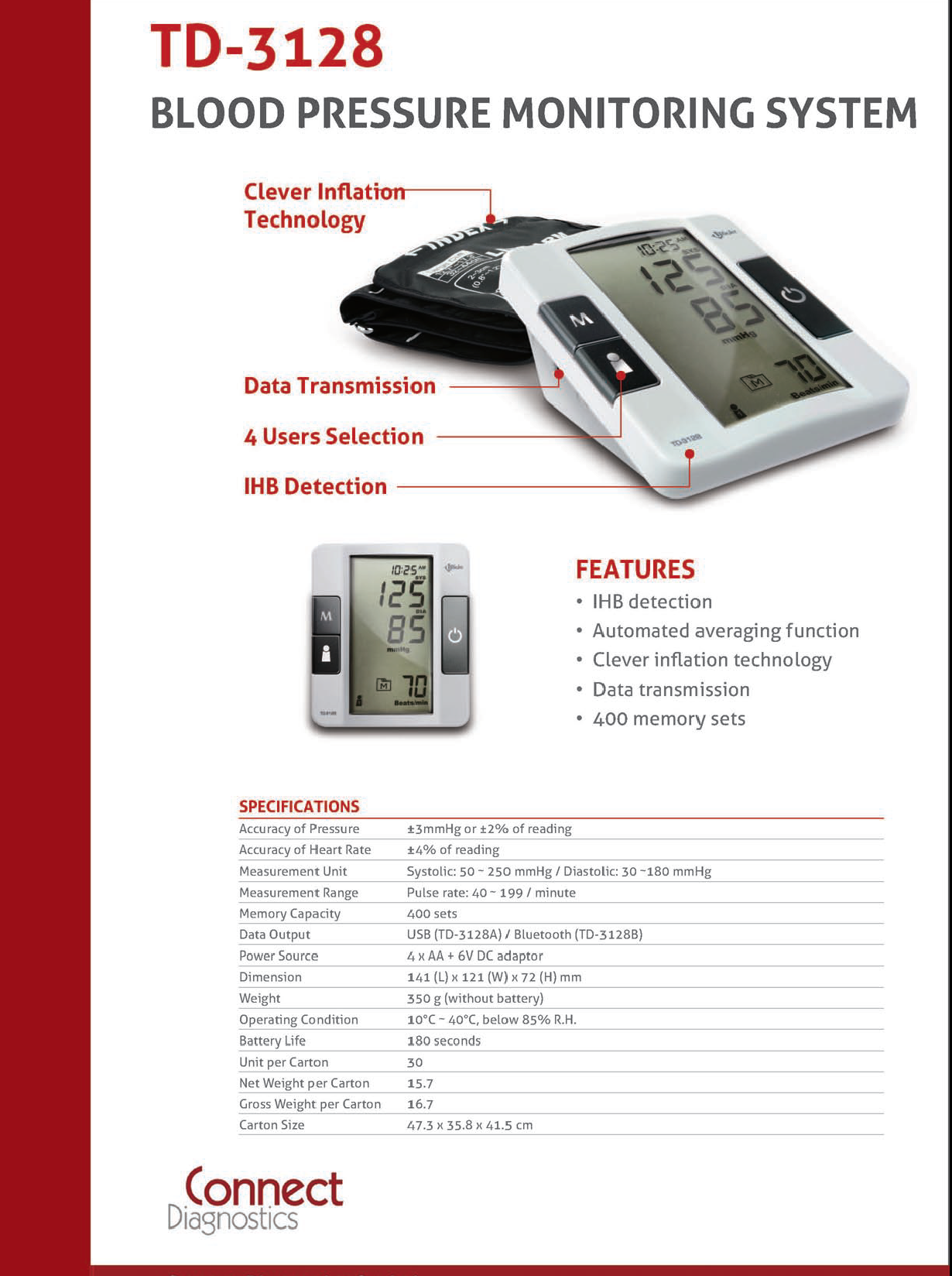


**Supplementary Table 1** – The first three most prescribed regimens at one-year follow-up.


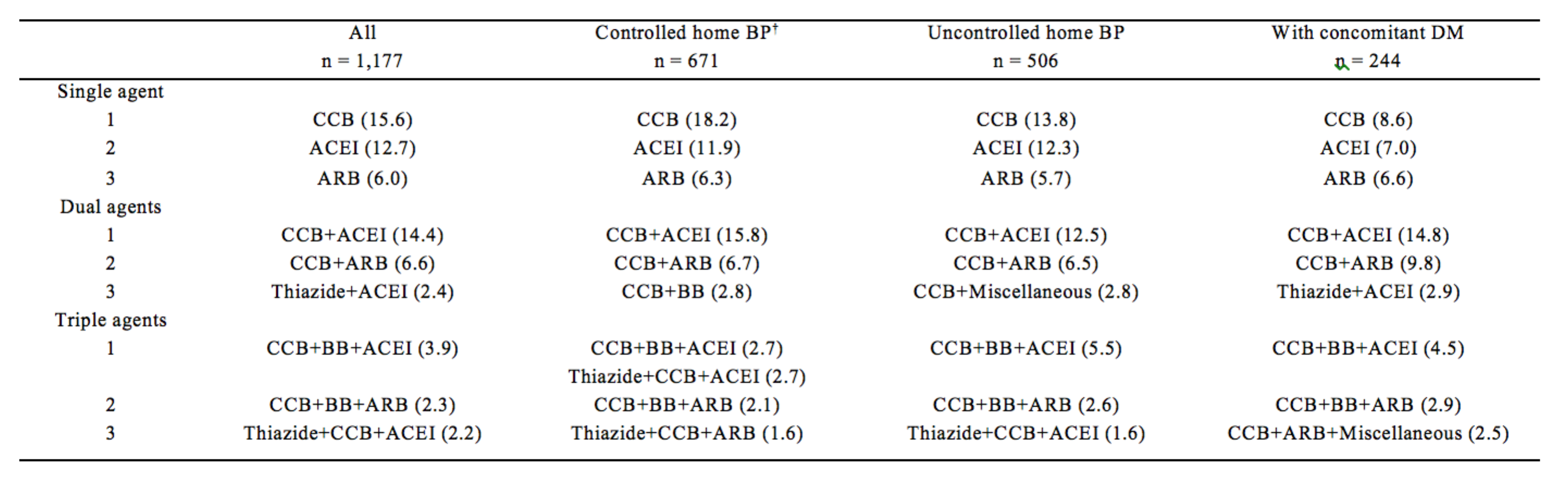


Values are (%). ^†^controlled home BP: patients who had home blood pressure data at one year < 135/85 mmHg.

CCB = calcium channel blocker; ACEI = angiotensin-converting enzyme inhibitor; ARB = angiotensin receptor blocker; BB = beta-adrenergic blocker; Thiazide = thiazide diuretic; DM = diabetes mellitus.

**Supplementary Table 2** – Prescribing frequency of each antihypertensive drug at one-year follow-up.

| Drug class | Generic name | All | Controlled  group | Uncontrolled group | With concomitant DM |
| --- | --- | --- | --- | --- | --- |
| CCB | Total | 741 (100) | 433 (100.0) | 308 (100.0) | 159 (100.0) |
|  | Amlodipine | 656 (88.5) | 402 (92.8) | 254 (82.5) | 130 (81.8) |
|  | Manidipine | 30 (4.0) | 13 (3.0) | 17 (5.5) | 12 (7.5) |
|  | Nifedipine | 22 (3.0) | 3 (0.7) | 19 (6.2) | 5 (3.1) |
|  | Diltiazem | 12 (1.6) | 3 (0.7) | 9 (2.9) | 7 (4.4) |
|  | Lercanidipine | 9 (1.2) | 5 (1.2) | 4 (1.3) | 2 (1.3) |
|  | Verapamil | 7 (0.9) | 3 (0.7) | 4 (1.3) | 2 (1.3) |
|  | Felodipine | 5 (0.7) | 4 (0.9) | 1 (0.3) | 1 (0.6) |
| ACEI | Total | 527 (100) | 285 (100.0) | 242 (100.0) | 107 (100.0) |
|  | Enalapril | 523 (99.2) | 283 (99.3) | 240 (99.2) | 106 (99.1) |
|  | Captopril | 3 (0.6) | 2 (0.7) | 1 (0.4) | 1 (0.9) |
|  | Quinapril | 1 (0.2) | 0 (0.0) | 1 (0.4) | 0 (0.0) |
| ARB | Total | 294 (100) | 163 (100.0) | 131 (100.0) | 84 (100.0) |
|  | Losartan | 268 (91.2) | 146 (89.6) | 122 (93.1) | 73 (86.9) |
|  | Valsartan | 11 (3.7) | 7 (4.3) | 4 (3.1) | 3 (3.6) |
|  | Irbesartan | 6 (2.0) | 6 (3.7) | 0 (0.0) | 3 (3.6) |
|  | Candesartan | 4 (1.4) | 3 (1.8) | 1 (0.8) | 2 (2.4) |
|  | Azilsartan | 2 (0.7) | 0 (0.0) | 2 (1.5) | 1 (1.2) |
|  | Telmisartan | 2 (0.7) | 1 (0.6) | 1 (0.8) | 1 (1.2) |
|  | Olmesartan | 1 (0.3) | 0 (0.0) | 1 (0.8) | 1 (1.2) |
| BB | Total | 286 (100) | 143 (100.0) | 143 (100.0) | 79 (100.0) |
|  | Atenolol | 174 (60.8) | 94 (65.7) | 80 (55.9) | 42 (53.2) |
|  | Metoprolol | 65 (22.7) | 25 (17.5) | 40 (28.0) | 20 (25.3) |
|  | Carvedilol | 21 (7.3) | 11 (7.7) | 10 (7.0) | 8 (10.1) |
|  | Propranolol | 21 (7.3) | 12 (8.4) | 9 (6.3) | 5 (6.3) |
|  | Bisoprolol | 5 (1.7) | 1 (0.7) | 4 (2.8) | 4 (5.1) |
| Diuretic | Total  Hydrochlorothiazide | 242 (100)  210 (86.8) | 137 (100.0)  127 (92.7) | 105 (100.0)  83 (79.0) | 60 (100.0)  49 (81.7) |
|  | Furosemide | 24 (9.9) | 6 (4.4) | 18 (17.1) | 9 (15.0) |
|  | Amiloride  Spironolactone | 7 (2.9)  1 (0.4) | 4 (2.9)  0 (0.0) | 3 (2.9)  1 (1.0) | 2 (3.3)  0 (0.0) |

Values are number (%)

Abbreviations as Supplementary Table 1

**Supplementary Figure 2**– Number of antihypertensive medications prescribed at baseline and at one year of follow-up between the controlled and uncontrolled HBP groups **at baseline**

**
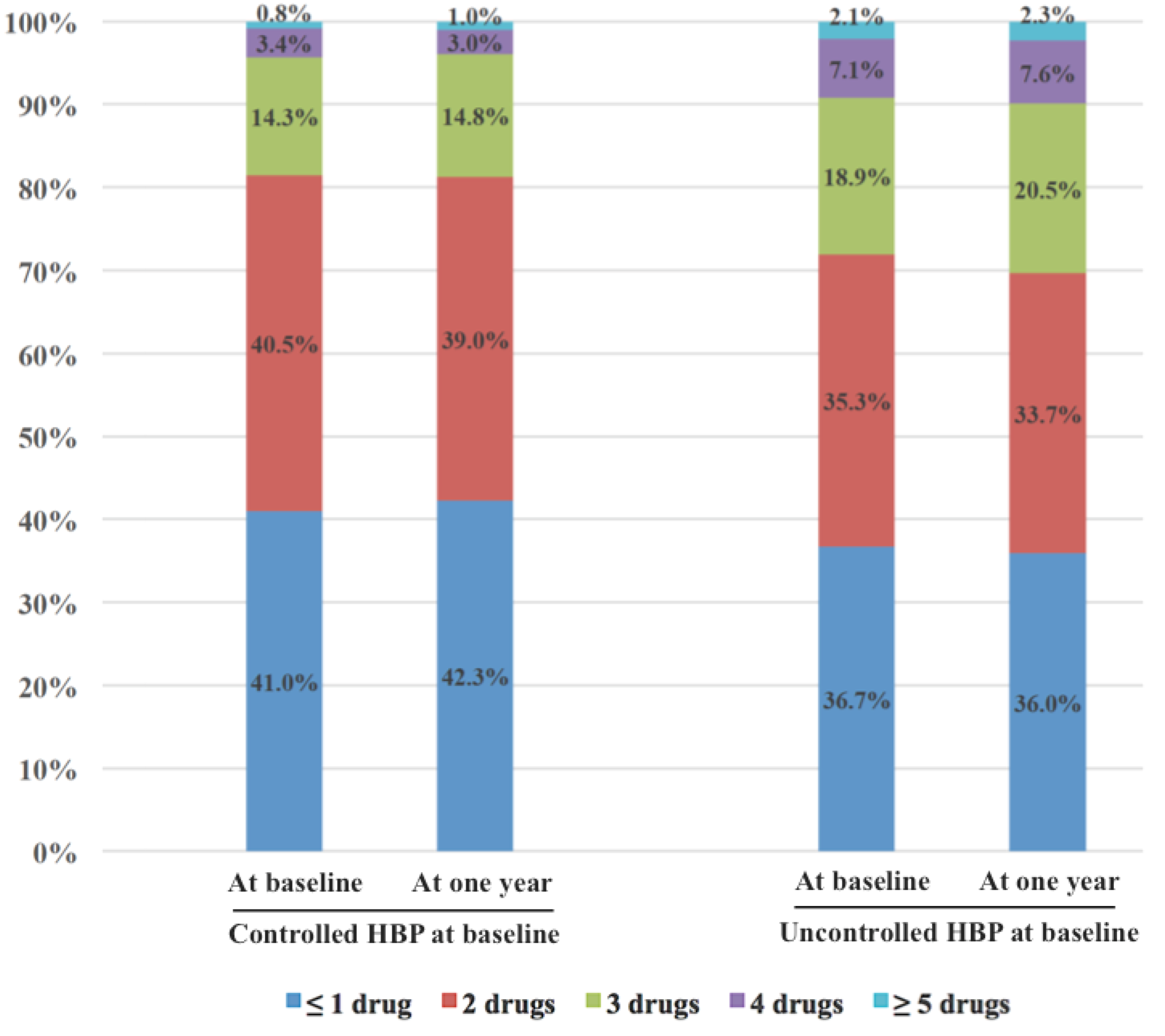
**

**Supplementary Figure 3**– Prescribing frequency of each antihypertensive drug class at

one year of follow-up in patients with concomitant diabetes.

DM = diabetes mellitus; CCB = calcium channel blocker; ACEI = angiotensin-converting enzyme inhibitor; ARB = angiotensin receptor blocker; BB = beta-adrenergic blocker; Thiazide = thiazide-type diuretic (hydrochlorothiazide, chlorthalidone); non-Thiazide = non-thiazide diuretic (amiloride, spironolactone, furosemide); miscellaneous = alpha-adrenergic blocker, hydralazine, clonidine, and methydopa.

**Supplementary Figure 4**– Prescribing frequency of each antihypertensive drug class at one-year follow-up according to the level of the hospital.

*p* = 0.961

***p* < 0.001**

***p* < 0.001**

***p* < 0.001**

*p* = 0.570

*p* = 0.640

*p* = 0.669

Abbreviations as Supplementary figure 3
